# Imaging Depiction of Hypoxic Pulmonary Vasoconstriction Using Dual-Energy CT

**DOI:** 10.7759/cureus.36551

**Published:** 2023-03-22

**Authors:** Joshua G Hunter, Priyanka Prajapati, Kaustav Bera, Aekta Gupta, Amit Gupta

**Affiliations:** 1 Radiology, Case Western Reserve University School of Medicine, Cleveland, USA; 2 Radiology, University Hospitals Cleveland Medical Center, Cleveland, USA; 3 Internal Medicine, Mercy Health - St. Elizabeth Youngstown Hospital, Youngstown, USA

**Keywords:** ventilation perfusion mismatch, ct (computed tomography) imaging, iodine perfusion map, hypoxic pulmonary vasoconstriction, dual-energy ct

## Abstract

In this article, we aim to highlight the utility of dual-energy computed tomography (DECT) in demonstrating imaging changes due to hypoxic pulmonary vasoconstriction (HPV). DECT allows detailed image reconstructions that have been shown to better characterize cardiothoracic pathologies, as compared to conventional CT techniques. DECT simultaneously detects two different X-ray energies, which enables generation of iodine density maps, virtual monoenergetic images, and effective atomic number maps (Z_eff_), among others. DECT has been shown to have utility in the assessment of benign versus malignant pulmonary nodules, pulmonary embolism, myocardial perfusion defects, and other conditions. Herein, we describe four cases of indeterminate pulmonary pathology when imaged with conventional CT in which subsequent use of DECT-derived image reconstructions demonstrated HPV as the underlying pathophysiological mechanism. The goal of this article is to understand the imaging appearance of HPV on DECT and discuss how HPV may mimic other causes of perfusion defects.

## Introduction

Dual-energy computed tomography (DECT) imaging technology, which utilizes two distinct X-ray energies in image acquisition, has proven to be advantageous over conventional CT imaging technology. The collection of attenuation measurements at two distinct X-ray energies in DECT enables further differentiation of materials than is possible with single-energy conventional CT [1].

A primary advantage of DECT is the ability to generate image reconstructions that require attenuation measurements at two X-ray energies, such as virtual monoenergetic images (VMI), iodine density maps and overlay images, and effective atomic number images (Z_eff_) [1,2]. By blending imaging datasets acquired at two energy levels, a VMI reconstruction allows for generation of images that mirror what would have been acquired at a specific, single-photon energy level. VMI reconstruction has been shown to improve image quality for DECT and to lower the necessary contrast agent dosage [3]. Iodine density maps can be generated with DECT since its attenuation characteristics are known and so attenuation generated by iodine can be isolated in the image [4]. Furthermore, iodine density maps can be overlayed with unenhanced images to more accurately correlate iodine levels with anatomic structures. Z_eff_ images, relying on DECT’s ability to estimate the effective atomic number of materials based on attenuation at two distinct photon energy levels, display the relative material atomic numbers in color at each voxel to assist in diagnosis of various pathologies [5].

Hypoxic pulmonary vasoconstriction (HPV) is an autoregulatory mechanism of the pulmonary vasculature that promotes ventilation/perfusion (V/Q) matching by inducing vasoconstriction in lung regions with poor alveolar ventilation. However, an impaired HPV response can arise in disease and generate systemic hypoxemia [6]. Distinguishing the etiology of persistent hypoxemia often requires distinction between various potential causes, such as pulmonary embolism (PE), mucous plugging, or impaired HPV, among others. This task can be complex when routine imaging does not reveal a pathophysiological mechanism. Utilization of DECT and its possible reconstructions can assist the radiologist in determining the pathophysiology and is particularly useful for the assessment of HPV given its invisibility on routine imaging. Iodine density maps and Z_eff_ reconstructions from DECT are especially valuable in assessing pulmonary perfusion defects and identifying lung regions impacted by HPV.

## Case presentation

Case 1

**Figure 1:**
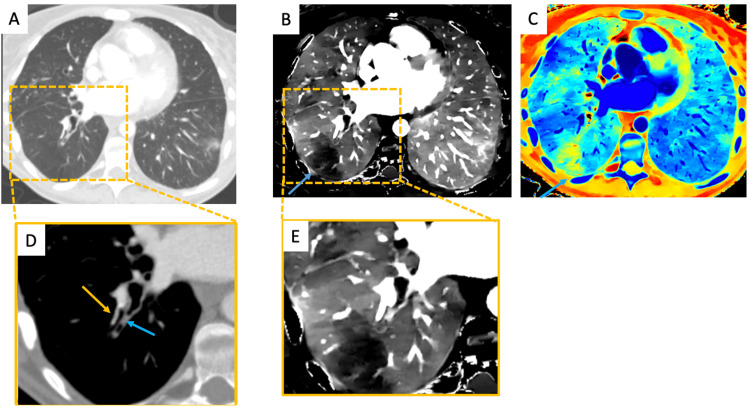
A 34-year-old male with a history of lung transplant presenting with acute right-sided chest pain with a D-dimer of 900 ng/mL. Initial axial CT scan of chest lung window setting (A) demonstrates no remarkable abnormalities. The corresponding iodine density map (B, E) and the Z_eff_ maps (C) show a peripheral wedge-shaped perfusion defect in the right lower lobe (blue arrow), mimicking pulmonary embolism. However, there is no evidence of pulmonary filling defect within the right lower lobe arterial branch supplying this region (tan arrow) on soft tissue window image (D) but a focal mucous plug within the segmental bronchus was detected (blue arrow), confirming that the perfusion defect is due to hypoxic pulmonary vasoconstriction. CT: Computed tomography

A 34-year-old male with a history of lung transplant presented with acute right sided chest pain. His D-dimer test was markedly elevated at 900 ng/mL and suggestive of PE when considered alongside his acute chest pain. But PE was not identified on initial contrast-enhanced CT imaging (Figures 1A, 1D). Subsequent acquisition of images on a DECT scanner enabled investigation with iodine density and Z_eff_ maps (Figures 1B, 1C, 1E). Both reconstructions demonstrated a wedge-shaped perfusion defect in the periphery of the right lower lobe with mucous plugging of the segmental bronchus and a patent pulmonary arterial branch. Hence, HPV was deemed the likely cause of his perfusion defect.

Case 2

**Figure 2:**
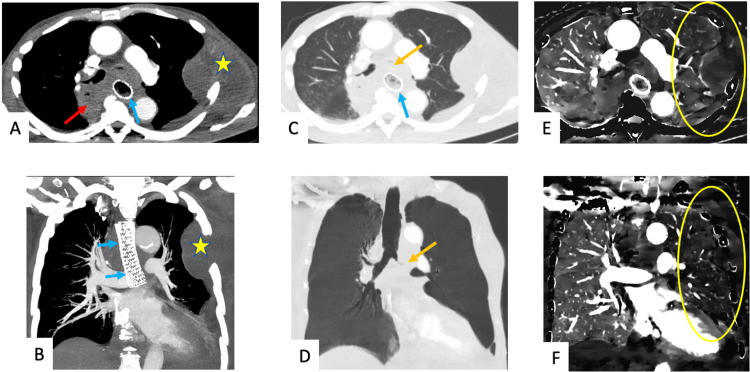
A 66-year-old male with advanced unresectable esophageal adenocarcinoma, status post stenting, presenting with shortness of breath. Axial and coronal CT images of the chest in soft tissue (A, B) and lung window (C, D) settings demonstrate a mass within the posterior mediastinum (red arrow), correlating with the patient's known primary esophageal malignancy with a stent in place (blue arrows) and associated complete obliteration/invasion of left principal bronchus (tan arrows). A left lateral chest wall metastatic mass is also visualized (yellow stars). On corresponding axial and coronal iodine density images (E, F), there is diffusely and significantly decreased perfusion throughout the left lung (yellow oval), likely secondary to hypoxic vasoconstriction from more proximal left mainstem bronchial occlusion. CT: Computed tomography

A 66-year-old male with advanced unresectable esophageal adenocarcinoma, for which an esophageal stent had been placed, presented with shortness of breath. CT angiography (CTA) was ordered given reasonable concern for PE. Patent pulmonary arteries were seen on CTA (Figures 2A, 2B). Images were subsequently acquired with a DECT scanner. Iodine density maps and quantification of iodine density demonstrated diffusely decreased perfusion throughout the left lung (Figures 2E, 2F). This perfusion defect was attributed to HPV secondary to invasion and complete obliteration of the left mainstem bronchus by the patient’s cancer (Figures 2C, 2D).

Case 3

**Figure 3:**
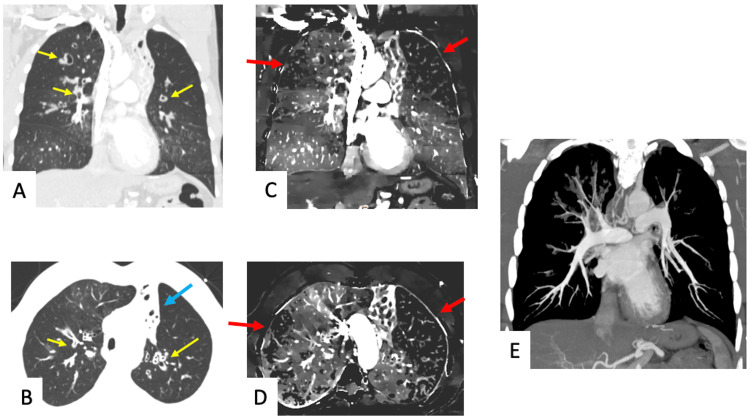
A 42-year-old male with history of chronic cystic fibrosis presenting with acute onset hemoptysis. Coronal and axial CT images in lung window setting (A, B) shows extensive upper lobe predominant bronchiectatic changes and multifocal mucous plugging (yellow arrows). Complete left upper lobe lung collapse is also visualized (blue arrow). The corresponding coronal and axial iodine density maps (C, D) demonstrate multiple wedge-shaped defects particularly in the left lung apex (red arrows), likely from hypoxic vasoconstriction given extensive mucous plugging and no pulmonary embolism as demonstrated by maximum intensity projection CT image (E). CT: Computed tomography

A 42-year-old male with a history of chronic cystic fibrosis presented with acute onset hemoptysis. Conventional CT angiography demonstrated extensive upper lobe predominant bronchiectatic changes and multifocal mucous plugging, along with complete left upper lobe collapse (Figures 3A, 3B). DECT-derived iodine density maps demonstrated multiple wedge-shaped perfusion defects, particularly in the left lung apex (Figures 3C, 3D). These perfusion defects are most likely due to HPV given extensive mucous plugging and the absence of PE (Figure 3E).

Case 4

**Figure 4:**
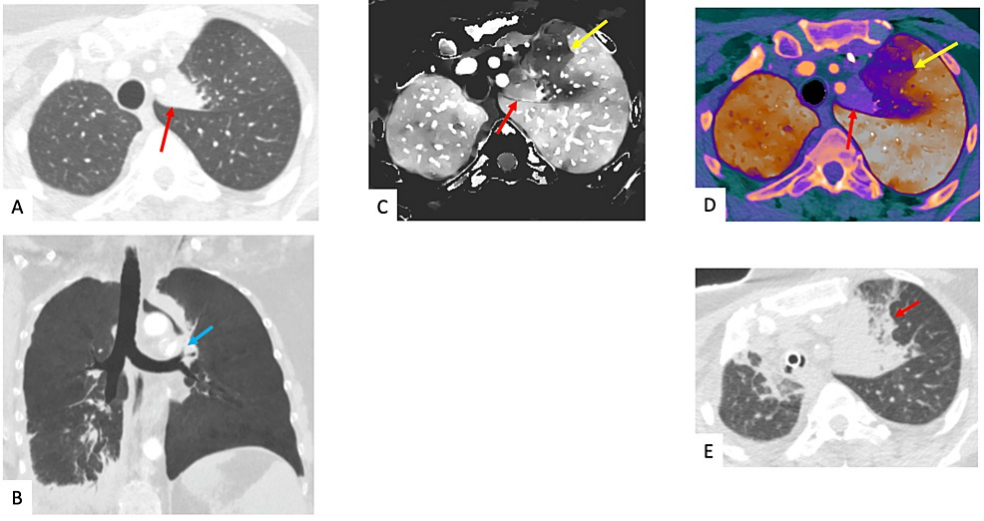
A 54-year-old female with a history of metastatic breast cancer presenting for follow-up chest CT imaging, status post radiation therapy to left mediastinal lymph node. Axial and coronal CT images in lung window setting (A, B) demonstrate a new severe stenosis/obliteration of left upper lobar segmental bronchus (blue arrow) with associated distal postobstructive atelectasis within the medial left upper lobe (red arrow). In corresponding iodine density maps (C) and overlay (D) images, there is demonstration of a geographical perfusion defect within the left upper lobe (yellow arrows) lateral to the homogeneously enhancing atelectasis (red arrows). This is likely due to hypoxic vasoconstriction and suggestive of the area at risk for impending volume loss/atelectasis. Subsequent follow-up chest CT scan (E) within three months confirms the above suspicion and demonstrates increasing left upper lobar atelectasis (red arrow), now also encompassing the area of the previously noted perfusion defect. CT: Computed tomography

A 54-year-old female with a history of metastatic breast cancer underwent radiation therapy to the left mediastinal lymph node. Follow-up chest CT imaging demonstrated stenosis and obliteration of the left upper lobe segmental bronchus with atelectasis distal to the stenotic obstruction (Figures 4A, 4B). The DECT-derived iodine density map and overlay image uncovered a perfusion defect in the left upper lobe near the site of atelectasis that places the area at risk for impending atelectasis and is most likely attributable to HPV (Figures 4C, 4D). A subsequent follow-up CT scan demonstrates increased atelectasis corresponding to the site of the HPV-induced perfusion defect (Figure 4E).

## Discussion

DECT is an imaging technology in which a dual-layer detector separately registers high- and low-energy photons to assess attenuation properties of materials at two distinct energy levels without requiring additional radiation exposure or prospective imaging protocol. Data obtained through DECT enable various spectral imaging reconstructions that are not possible with conventional CT technology.

Application of DECT-derived image reconstructions to evaluate for HPV has shown promising results [7]. In this article, we further enumerate the advantages through our cases that encompass several clinical scenarios in which DECT imaging was used to improve the characterization of perfusion defects and unveil the responsible pathophysiological mechanism to be HPV that is invisible on conventional CT imaging. Additionally, our cases exemplify that HPV is an important mimicker of other pathologies that cause perfusion defects. This case series thus highlights how HPV appears on DECT imaging and demonstrates how it mimics several pathologies that also cause perfusion defects.

The first case discusses a patient with a history of lung transplant and suspicion of PE, given acute onset chest pain coupled with an elevated D-dimer. Although conventional CT imaging was unremarkable, DECT demonstrated a wedge-shaped perfusion defect suggestive of PE. However, close evaluation demonstrated the mucous plug and resultant HPV as the cause of decreased perfusion. With DECT increasingly being used for the assessment of PE-related perfusion defects, this case highlights the importance of using a combination of conventional CT images and DECT reconstructions to reach the correct diagnosis, which in this case was HPV related to mucous plugging. The second case shows how HPV can cause severe perfusion defects, equating to complete vascular shutdown of the left lung in this patient, which are commonly attributed to other pathologies. Therefore, it is important to keep HPV in the differential for diffuse perfusion defects and to appreciate how DECT enables detection of HPV. In the third case, routine imaging revealed mucous plugging in a patient with cystic fibrosis presenting with acute hemoptysis while DECT imaging revealed HPV as the pathophysiological mechanism underlying the patient’s presentation. The ability to confidently determine ongoing HPV with DECT imaging adds clinical value by limiting the need for further investigation. Finally, the fourth case highlights how DECT may be used to evaluate the full extent of an abnormality detected on routine CT and how findings on DECT may have the potential to predict impending derangements secondary to a correctable airway obstruction.

## Conclusions

In this article, we have discussed and enumerated through various cases the ability of DECT to capture attenuation measurements at two distinct photon energy levels, which in turn provides unique advantages over conventional CT that may be leveraged to enhance evaluation of cardiothoracic pathologies. We have shown how DECT reconstructions can be used in the assessment of perfusion defects, and in particular to uncover HPV-induced perfusion defects that cannot be visualized on conventional CT. Correctly identifying that HPV is the underlying mechanism behind perfusion defects, rather than a more emergent pathology like PE, has clinical relevance in potentially limiting further diagnostic testing and guiding appropriate clinical management. As DECT methods become increasingly commonplace in clinical settings, radiologists must understand how HPV presents on DECT imaging and how it mimics other pathologies that cause perfusion defects.
